# Genome-Wide Association Study Reveals Novel Loci and Candidate Genes for Birth Weight in Pigs

**DOI:** 10.3390/ani15060825

**Published:** 2025-03-13

**Authors:** Jiajia Liu, Yue Zeng, Yu Tian, Linghua Cheng, Wenchao Li, Shunfeng Cheng, Junjie Wang, Lan Li

**Affiliations:** 1 Key Laboratory of Animal Reproduction and Biotechnology in Universities of Shandong, College of Animal Science and Technology, Qingdao Agricultural University, Qingdao 266109, China; jiajialiu@qau.edu.cn (J.L.); zengyuezy0220@163.com (Y.Z.); leon.tianyu@hotmail.com (Y.T.); sfcheng@qau.edu.cn (S.C.); junjieseven0717@163.com (J.W.); 2 Beijing Dabeinong Technology Group Co., Ltd., Beijing 100194, China; chenglinghua1990@163.com; 3 Zhaoqing Dabeinong Agricultural and Pastoral Food Co., Ltd., Zhaoqing 526000, China; fly3xun@aliyun.com

**Keywords:** GWAS, birth weight, SNP, *MARCHF11*, pig

## Abstract

Birth weight is a key economic trait in pig breeding, affecting pre-weaning survival, growth performance, and overall production efficiency. However, the genetic factors underlying birth weight remain incompletely understood. This study aimed to identify genetic variants associated with birth weight in pigs through a genome-wide association study (GWAS) using 50K SNP genotyping data from 1125 Landrace pigs. Seven significant SNPs linked to birth weight were identified, along with 13 potential candidate genes. To validate these findings, the study population was expanded to include 998 Yorkshire pigs, totaling 2123 individuals. Validation identified two novel SNPs on chromosomes 1 and 16, as well as the previously unreported gene *MARCHF11*, which was associated with birth weight. These findings enhance the understanding of the genetic architecture underlying birth weight and provide potential targets for molecular marker-assisted selection (MAS). The results offer valuable insights into breeding strategies aimed at improving piglet survival rates and production efficiency. This study underscores the utility of GWAS in identifying key genetic loci for economically important traits in livestock populations.

## Simple Summary

Birth weight is a key trait in pig breeding, influencing survival and growth. However, its genetic basis is not fully understood. This study aimed to identify genetic factors associated with birth weight using a genome-wide association study on pigs. The research uncovered several significant loci and the novel gene *MARCHF11* related to birth weight. These findings provide insights into the genetic regulation of birth weight and offer potential markers for improving breeding strategies. By enhancing selection for birth weight, this study can help increase piglet survival and production efficiency, benefiting the swine industry.

## 1. Introduction

Birth weight is a fundamental economic trait in pig breeding, significantly influencing early survival, growth performance, and overall production efficiency. It is a critical determinant of pre-weaning survival rates and plays a vital role in the subsequent growth and development of pigs [1,2]. However, despite its economic and biological importance, the genetic basis underlying birth weight remains incompletely understood, posing challenges to its enhancement through selective breeding.

In recent years, genome-wide association studies (GWASs) have become a powerful tool for identifying genetic variants associated with complex traits in pigs [3,4]. By utilizing high-density SNP genotyping arrays and sophisticated statistical models [5,6], GWASs have provided valuable insights into the genetic architecture of various economically important traits, such as growth traits [7,8,9,10,11,12], reproduction traits [13,14,15], and meat quality [4,16,17,18]. For the birth weight of pigs, several studies have reported genetic loci associated with this trait [3,19,20]. For instance, two significant SNPs on chromosomes 1 (97,745,041 bp) and 4 (112,031,589 bp) were identified to be associated with birth weight in Yorkshire and Landrace pigs [21]. Another study recognized four birth weight-associated genomic regions on chromosomes 2, 4, 5, and 7 through a GWAS from three different pure breeds, explaining 8.42% of the genetic variance for birth weight [1]. Moreover, 11 SNPs linked to litter birth weight were carried out on two pig breeds (Yorkshire and Landrace) [22]. Previous studies have also identified candidate genes using GWASs, such as *SMAD4*, *RPS6KA2*, *CAMK2A*, and *NDST1*; these genes are mainly enriched in the calcium signaling, ovarian steroidogenesis, and GnRH signaling pathways [23]. What is more, candidate genes such as *APPL2*, *TGFB1*, *MACROH2A1*, and *SEC22B* have been reported to be relevant to birth weight [1]. Despite these findings, results across studies have been inconsistent, and many genetic factors contributing to birth weight remain unexplored. These discrepancies likely stem from differences in study populations, statistical models, and environmental influences.

In order to understand the genetic mechanisms underlying birth weight in pigs, this study aimed to systematically elucidate its genetic basis and advance breeding applications through an integrated approach combining genome-wide association study and cross-population validation strategies.

## 2. Materials and Methods

### 2.1. Data Collection and Quality Control

Birth weight records were collected between 2023 and 2024 from commercial pig farms in Puzhehei, Yunnan, China, encompassing two populations: 1145 Landrace pigs (LL) and 1000 Yorkshire pigs (YY). The inclusion criteria for the pigs were healthy newborn pigs verified by veterinarians, with a balanced gender ratio. Environmental and animal factors, including herd (Farm 1 and Farm 2), year (2023 and 2024), season (spring, summer, autumn, and winter), parity (1, 2, 3, and ≥4), and sex (female and male), were recorded alongside the phenotypic data. To ensure data accuracy, records exceeding ±3 standard deviations from the mean were excluded. After quality control, 1125 and 998 records were retained for the two populations, resulting in a total of 2123 valid records for further analysis.

### 2.2. Statistical Analysis of Environmental and Animal Factors

The effects of environmental and animal factors on birth weight were assessed using a least-squares means (LSM) linear model with analysis of variance (ANOVA). The R package emmeans (https://cran.r-project.org/web/packages/emmeans/index.html, accessed on 2 October 2024) was used to fit and adjust the model. Only those factors significantly affecting birth weight in the Landrace pig population (*p* < 0.05) were included as fixed effects in the subsequent genome-wide association study model. To visualize the impact of breeds or environmental factors on birth weight in the pig population, boxplots were generated using the ggplot2 package in R [24].

### 2.3. Genotyping and Quality Control

Ear tissue samples were collected from the pigs for genomic DNA extraction, using the standard phenol-chloroform method to ensure high quality and quantity of DNA [25,26]. Genotyping was performed using the 50K “Zhongxin No. 1” chip (Compass Biotechnology, Beijing, China), designed to assess genetic variation across the pig genome. The raw genotypic data were processed and quality-controlled using PLINK v1.9 [27]. SNPs with a genotyping rate < 95%, minor allele frequency (MAF) < 0.01, or missing map information were excluded. Only SNP loci on autosomes 1 to 18 were retained. Similarly, individuals with a genotyping rate < 95% were removed from the analysis. Following quality control, a total of 45,402 high-quality SNPs and 1145 samples in the Landrace population were retained for the GWAS.

### 2.4. Genome-Wide Association Analysis

Genome-wide association analysis was performed using the Fixed and Random Model Circulating Probability Unification (FarmCPU) method, implemented with the R package rMVP [28]. This approach iteratively alternates between fixed- and random-effect models to identify genetic variants associated with birth weight in pigs. The fixed-effect model included the first three principal components (PCA = 3) to account for population structure as well as environmental and animal factors, including herd, year, season, parity, and sex. The model is described as follows:

*y* = *Xb* + *M_i_b_i_* + *S_j_d_j_* + *e*

where *y* is the observation vector; *X* is the matrix of fixed effects, including principal components and environmental covariates; *M_i_* is the fixed-effect genotype matrix for each SNP; *b* and *b_i_* are the design matrices for the fixed effects and SNP genotypes; *S_j_* represents the jth SNP to be analyzed, and *d_j_* is its corresponding effect. The residual error term *e* is assumed to follow a normal distribution *e~N* (0, *Iσ_e_*^2^).

The random-effect model was applied to account for genetic relationships between individuals and is given by the following:

*y* = u + *e*

where *u* represents the genetic effect, assumed to follow a normal distribution *u*∼*N* (0, *Kσ_u_*^2^), with *K* being the genetic relationship matrix derived from SNP genotyping data. The residual error term *e* remains the same as in the fixed-effect model.

The analysis alternated between the fixed- and random-effect models in an iterative process, refining the detection of significant SNPs. To control for multiple testing, the Bonferroni correction was applied, adjusting the significance threshold to *p* < 1.1 × 10^−6^ (0.05/number of effective SNPs). SNPs with a *p*-value < 1.0 × 10^−4^ were considered as candidate loci, ensuring the reliability and robustness of the findings.

Quantile–quantile (Q–Q) plots were generated to compare the observed −log_10_ (*p*-value) distribution for each SNP with the expected distribution under the null hypothesis for each trait. Manhattan plots were constructed to visualize significant SNP associations across chromosomes and traits. All visualizations were produced using the R package rMVP [28]. Given the sample size and research objectives, interaction effects were not included in this stage of analysis. Future studies will explore potential interaction effects through expanded cohorts.

### 2.5. Candidate Gene Functional Annotation

Significant and candidate SNPs identified from the genome-wide association analysis were annotated using the pig reference genome (*Sscrofa11.1* assembly) by SnpEff v5.2c [29] to determine their associated genes. Genes located within these SNPs were designated as candidate genes potentially influencing birth weight in pigs.

Functional enrichment analysis of the candidate genes was performed using the Metascape platform (https://metascape.org, accessed on 10 October 2024), including Gene Ontology (GO) and pathway analyses to elucidate their biological roles. Additionally, protein–protein interactions (PPIs) involving the candidate genes were analyzed and visualized using the IntAct Molecular Interaction Database (https://www.ebi.ac.uk/intact, accessed on 10 November 2024), providing insights into the molecular networks underlying birth weight regulation.

Expression profiles of the candidate genes across various pig tissues were retrieved from the PigGTEx database (http://piggtex.farmgtex.org/, accessed on 19 November 2024). The expression data were visualized using the R package ggplot2 [24], enabling a comprehensive understanding of the spatial and functional expression patterns of the candidate genes.

### 2.6. Validation of SNP Effects and Impacts on Birth Weight

The associations between the three genotypes of each of the identified SNPs and birth weight were evaluated in both the discovery population (LL) and the validation population (LL + YY) using two complementary approaches to ensure robust and accurate results. Firstly, a Kruskal–Wallis (KW) test was conducted to assess the impact of different genotypes at each locus on birth weight. Violin plots were generated using the ggplot2 package in R [24] to visualize the results, with significant differences between genotypes clearly annotated. Secondly, an LSM approach was applied, combined with Tukey’s Honest Significant Difference (HSD) test, to evaluate pairwise differences between genotypes. The LSM model included environmental and animal factors such as breed, herd, year, season, parity, and sex as fixed effects, adjusted using the R package emmeans. Only factors with a significant impact on birth weight (*p* < 0.05) were retained in the final model (Table 1). SNP loci were considered novel and significantly associated with birth weight if they exhibited consistent significant effects across both the test and validation populations using both analytical methods.

## 3. Results

### 3.1. Descriptive Statistics of Birth Weight and Environmental Influences in Pigs

**Figure 1:**
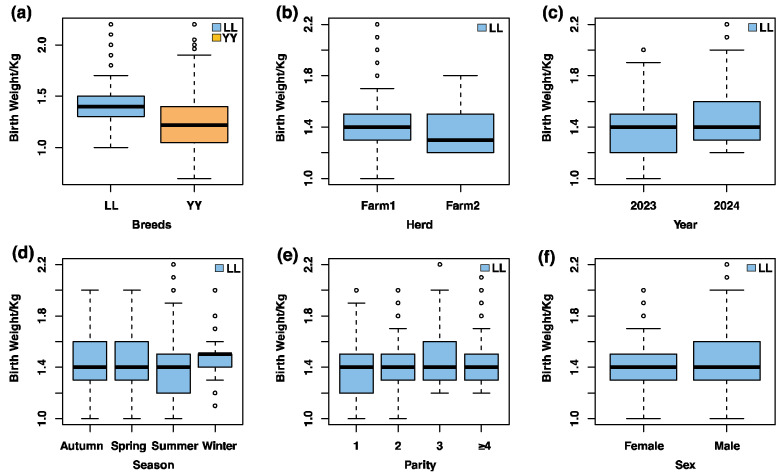
Boxplot of factors influencing birth weight in pigs. LL represents Landrace pigs, and YY represents Yorkshire pigs: (**a**) effects of breed on birth weight in the total population (LL and YY); (**b**) effects of herd on birth weight in the Landrace pigs; (**c**) effects of year on birth weight in the Landrace pigs; (**d**) effects of season on birth weight in the Landrace pigs; (**e**) effects of parity on birth weight in the Landrace pigs; (**f**) effects of sex on birth weight in the Landrace pigs. Circles indicate values beyond Q3 + 1.5 × IQR or below Q1 − 1.5 × IQR, where Q1, Q3, and IQR stand for the first quartile, third quartile, and interquartile range respectively.

After excluding outliers, birth weight phenotypic data remained for 2123 records from the two pig populations: Landrace (*n* = 1125) and Yorkshire (*n* = 998). The average birth weight for Landrace pigs was 1.42 ± 0.20 kg, while for Yorkshire pigs, it was 1.22 ± 0.25 kg (Table 1). A highly significant difference in birth weight between the Landrace and Yorkshire breeds was observed (*p* < 0.001, Table 2 and Figure 1a), reflecting breed-specific variations that may be influenced by both genetic and environmental factors.

The Landrace population was selected for the GWAS to identify SNPs and candidate genes associated with birth weight. These findings were subsequently validated in the total population of Landrace and Yorkshire pigs. To examine the impact of environmental and animal factors on birth weight, an LSM model was employed. Significant effects on birth weight were found for herd (Farm 1 and Farm 2), year (2023 and 2024), season (spring, summer, autumn, winter), parity (1, 2, 3, and ≥4), and sex (male and female) in the Landrace population (Table 2).

To examine the influence of environmental and animal factors, we assessed the effects of herd, year, season, parity, and sex on birth weight. Significant effects were observed for all these environmental factors. For example, birth weight in the winter (1.46 ± 0.14 kg) was significantly higher than in the summer (1.39 ± 0.20 kg), and male pigs had a significantly higher average birth weight (1.44 ± 0.18 kg) compared to females (1.40 ± 0.19 kg) (Figure 1b–f). These results underscore the importance of accounting for environmental influences in genetic studies of birth weight.

### 3.2. Genome-Wide Association Study in Landrace Pigs

**Figure 2:**
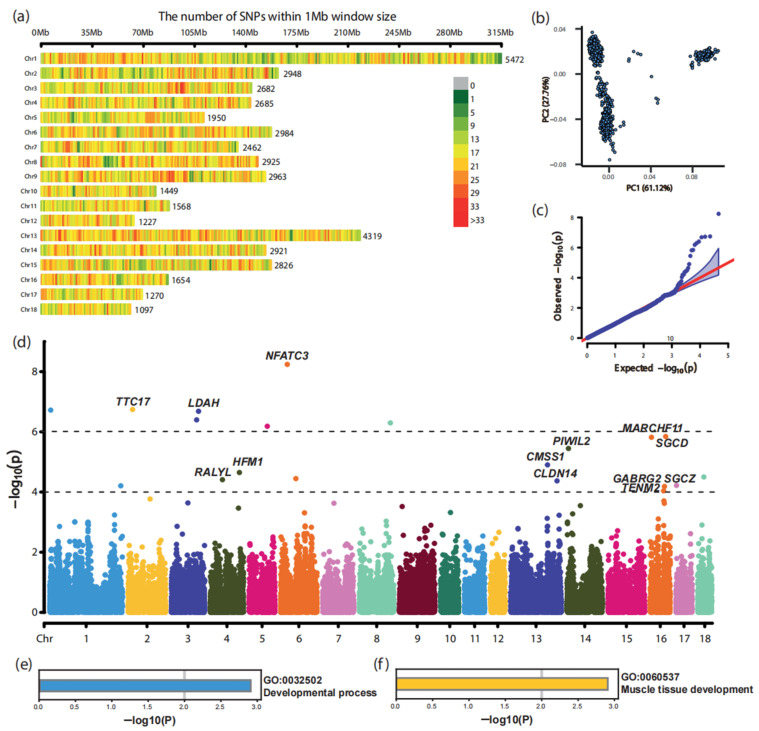
GWAS identified SNPs and candidate genes affecting birth weight in Landrace pigs. (**a**) Genomic distribution of SNP loci that passed quality control; (**b**) principal component analysis (PCA) of the Landrace population; (**c**) Q-Q plot assessing the reliability of the GWAS, the red line is the theoretical quantile line; (**d**) Manhattan plot showing the GWAS results; (**e**) functional enrichment of candidate genes in GO parent biological processes; (**f**) functional enrichment of candidate genes in GO biological processes.

A total of 45,402 high-quality SNPs and phenotype–genotype data from 1125 Landrace pigs were analyzed to identify genomic loci and candidate genes associated with birth weight. The SNPs were evenly distributed across the genome, with chromosome 1 having the highest number of SNPs (5472) and chromosome 18 the lowest (1097) (Figure 2a). Variance component analysis based on the genotype data estimated a heritability of 0.32 for birth weight in the Landrace population, suggesting that approximately 32% of the variation in birth weight is attributable to genetic factors, with the remaining variation influenced by environmental factors.

Using the FarmCPU method [1], which integrates fixed- and random-effect models iteratively, seven SNPs were identified as being significantly associated with birth weight at the genome-wide significance threshold (*p* < 1.1 × 10^−6^), while an additional 13 SNPs were detected as suggestive loci (*p* < 1.0 × 10^−4^) (Figure 2c,d).

Annotation using the *Sus scrofa* genome assembly 11.1 revealed that the identified SNPs were located within 13 protein-coding genes, including *LDAH*, *NFATC3*, *CMSS1*, *MARCHF11*, *GABRG2*, etc. (Table 3). These genes were identified as candidate genes, mainly distributed across chromosomes 3, 4, 13, and 16.

Furthermore, functional enrichment analysis of these candidate genes revealed their significant involvement in critical biological processes of “GO:0032502 developmental process” and “GO:0060537 muscle tissue development” (Figure 2e,f). These findings imply that the identified genes may play essential roles in regulating birth weight by influencing muscle growth and developmental pathways.

### 3.3. Validation of GWAS-Identified SNPs for Birth Weight Across Populations

To validate the effects of the 20 candidate SNPs identified in the GWAS analysis, we employed the KW test to assess genotype-based differences in birth weight across the discovery (LL) and validation (LL + YY) populations. Among these loci, 10 SNPs exhibited significant genotype-dependent effects on birth weight in both populations.

**Figure 3:**
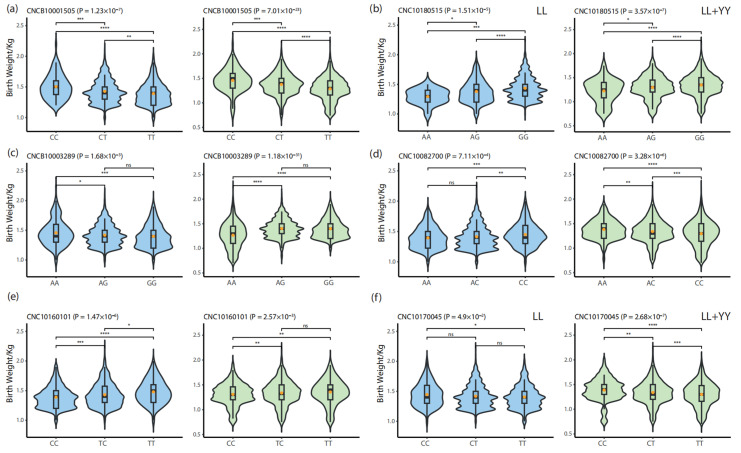
Violin plots illustrating birth weight distributions across three genotypes for each locus assessed using the KW test. Blue indicates the discovery population (LL), and green represents the validation population (LL + YY). (**a**) CNCB10001505; (**b**) CNC10180515; (**c**) CNCB10003289; (**d**) CNC10082700; (**e**) CNC10160101; (**f**) CNCB10009475. Significance markers: **** represents *p* ≤ 0.0001, *** represents *p* ≤ 0.001, ** represents *p* ≤ 0.01, * represents *p* ≤ 0.05, and ns represents no statistical significance (*p* ≥ 0.05).

Notably, two loci, CNCB10001505 (307,515,291 bp on chromosome 1) and CNC10180515 (26,725,722 bp on chromosome 18), showed significant differences between all three genotypes, with a consistent trend across both populations. At CNCB10001505, pigs with the CC genotype had the highest birth weight, followed by the CT and TT genotypes. In the discovery population, the mean birth weights for the CC, CT, and TT genotypes were 1.50 ± 0.20 kg, 1.43 ± 0.19 kg, and 1.39 ± 0.18 kg, respectively. In the validation population, the corresponding birth weights were 1.46 ± 0.24 kg, 1.38 ± 0.22 kg, and 1.29 ± 0.24 kg. Similarly, at CNC10180515, pigs with the GG genotype exhibited the highest birth weight, followed by the AG and AA genotypes. In the discovery population, the mean birth weights for the GG, AG, and AA genotypes were 1.43 ± 0.19 kg, 1.38 ± 0.19 kg, and 1.29 ± 0.13 kg, respectively, while in the validation population, these values were 1.35 ± 0.24 kg, 1.30 ± 0.24 kg, and 1.23 ± 0.25 kg (Figure 3a,b).

Two additional loci, CNCB10003289 (52,145,174 bp on chromosome 4) and CNC10082700 (133,591,911 bp on chromosome 8), also demonstrated significant genotype effects on birth weight but showed opposite trends between the discovery and validation populations. At CNCB10003289, in the discovery population, the birth weight trend was AA (1.46 ± 0.21 kg) > AG (1.42 ± 0.18 kg) > GG (1.40 ± 0.19 kg), whereas in the validation population, the trend reversed to AA (1.27 ± 0.26 kg) < AG (1.40 ± 0.19 kg) = GG (1.40 ± 0.19 kg). For CNC10082700, in the discovery population, the birth weight trend was AA (1.39 ± 0.18 kg) < AC (1.41 ± 0.20 kg) < CC (1.45 ± 0.19 kg), while in the validation population, the trend was reversed to AA (1.38 ± 0.20 kg) > AC (1.34 ± 0.23 kg) > CC (1.30 ± 0.26 kg) (Figure 3c,d).

The remaining six loci (CNC10160101, CNCB10009475, CNC10032128, CNC10060592, CNC10061395, and CNC10170045) displayed significant differences in birth weight between at least one homozygous genotype and the heterozygous genotype. For these loci, the relationship between genotype and birth weight followed a consistent trend across both populations. For instance, at CNC10160101, in the discovery population, the birth weights for the CC and TC genotypes were 1.39 ± 0.18 kg and 1.43 ± 0.20 kg, respectively, while in the validation population, the corresponding values were 1.31 ± 0.23 kg and 1.34 ± 0.25 kg (Figure 3e,f).

### 3.4. Key SNPs and MARCHF11 Gene Associated with Pig Birth Weight

**Figure 4:**
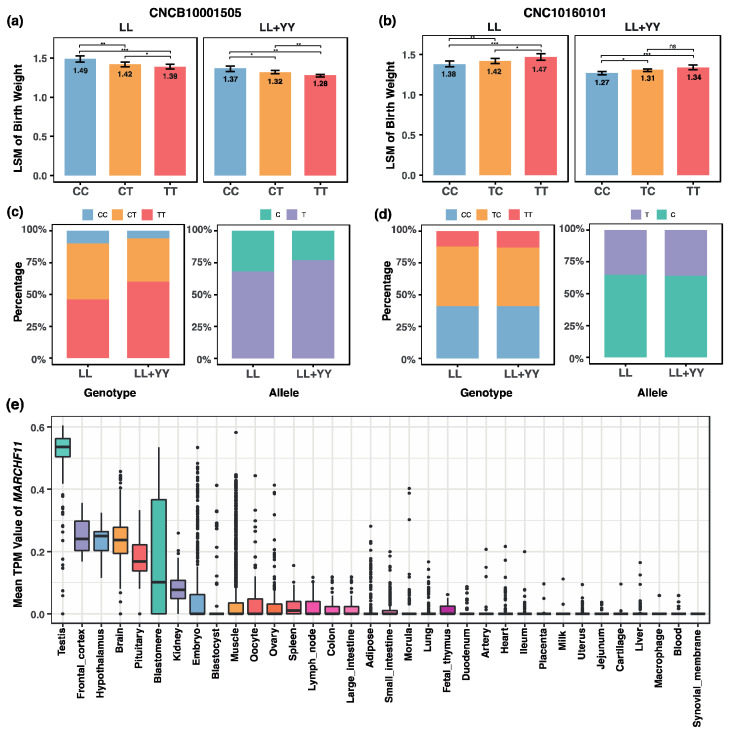
Key SNPs and *MARCHF11* gene associated with birth weight in the discovery (LL) and validation (LL + YY) populations. (**a**,**b**) Least-squares means of birth weight across three genotypes for CNCB10001505 and CNC10160101; (**c**,**d**) phenotype and allele percentages for CNCB10001505 and CNC10160101; (**e**) gene expression of the *MARCHF11* gene in different pig tissues. *** represents *p* ≤ 0.001, ** represents *p* ≤ 0.01, * represents *p* ≤ 0.05.

We further investigated the genotype-based differences in birth weight at SNP loci identified through the GWAS using the LSM model, which accounted for breed and environmental factors as fixed effects. After adjustment, eight SNPs remained significantly associated with birth weight in both the discovery and validation populations. Notably, two loci—CNCB10001505 and CNC10160101—were consistently identified as significant across both the KW and LSM validation methods, emphasizing their strong and reproducible associations with birth weight (Figure 4a,b).

At CNCB10001505, the CC genotype was associated with the highest birth weight (1.49 ± 0.02 kg), followed by CT (1.42 ± 0.01 kg) and TT (1.39 ± 0.02 kg). Similarly, at CNC10160101, the TT genotype exhibited the highest birth weight (1.47 ± 0.02 kg), followed by TC (1.42 ± 0.01 kg) and CC (1.38 ± 0.02 kg). Despite their favorable effects on birth weight, the advantageous genotypes—TT at CNCB10001505 and CC at CNC10160101—were found to occur at relatively low frequencies in both the discovery population (10% and 6%, respectively) and the validation population (12% and 13%, respectively). Furthermore, the favorable alleles (T and C) at these loci were also underrepresented, with frequencies of 32% and 35% in the discovery population and 23% and 36% in the validation population. In different pig tissues, MARCHF11 has the highest expression level in the testis (Figure 4c–e).

## 4. Discussion

The identification of effective genetic markers associated with birth weight in pigs is crucial for improving piglet survival rates and overall production efficiency [30]. In this study, we identified several candidate SNPs associated with birth weight through a GWAS in a Landrace pig population. To validate these findings, we employed the KW test and an LSM model, which confirmed the significant effects of these loci across both the discovery and validation populations. Notably, two loci—CNCB10001505 and CNC10160101—were consistently significant across both validation methods, demonstrating their robust association with birth weight. This multi-step validation approach not only enhances the reliability of our findings but also underscores the potential applicability of these SNPs in cross-breeding strategies. Future studies should focus on functional validation and further exploration of these loci to fully understand their role in pig growth and development.

One of the key findings of this study is the identification of the *MARCHF11* gene, located near the significant SNP CNC10160101 on chromosome 16. *MARCHF11* is a member of the MARCH family of E3 ubiquitin ligases, which are involved in protein ubiquitination—a process critical for regulating protein stability, cellular signaling, and embryonic development. Ubiquitination plays a pivotal role in early embryonic development, particularly in cell proliferation and differentiation, suggesting that *MARCHF11* may influence birth weight by modulating these processes. This finding aligns with previous studies that have highlighted the importance of ubiquitination pathways in fetal growth and development [1,2]. Further functional studies, such as gene knockout or overexpression experiments, are needed to validate the role of *MARCHF11* in regulating birth weight.

In addition to *MARCHF11*, we identified several other candidate genes, including *NFATC3*, *SGCD*, and *SGCZ*, which are known to be involved in muscle and skeletal development. These genes may directly influence fetal body composition and growth, thereby affecting birth weight. Functional enrichment analysis revealed that these genes are significantly involved in biological processes related to muscle tissue development and embryonic growth, further supporting their potential roles in birth weight regulation. For example, *NFATC3* has been implicated in muscle development and differentiation, while *SGCD* and *SGCZ* are associated with skeletal muscle function [8,22]. These findings are consistent with previous GWASs that have identified genes involved in muscle development as key regulators of birth weight in pigs [21,23]. In contrast, genes involved in metabolic processes, such as *LDAH* and *CMSS1*, as well as those involved in developmental regulation, including *HFM1*, *PIWIL2*, and *TENM2*, may impact birth weight indirectly by modulating embryonic development, cell proliferation, or energy metabolism.

Our results also highlight the importance of considering environmental factors in genetic studies of birth weight. We observed significant effects of herd, year, season, parity, and sex on birth weight, with male pigs generally having higher birth weights than females. Additionally, birth weight varied across seasons, with winter pigs showing the highest birth weights. These findings are consistent with previous studies that have demonstrated the influence of environmental factors on birth weight in pigs [2,7]. Environmental conditions such as temperature, nutrition, and management practices can significantly impact fetal growth and development, and these factors should be carefully considered in future breeding programs to optimize birth weight.

The discrepancies between our findings and those of previous GWASs may be attributed to differences in study populations, statistical models, and environmental factors. For example, while we identified significant associations with SNPs on chromosomes 1, 16, and 18, other studies have reported associations on chromosomes 2, 4, 5, and 7 [1,21]. These inconsistencies highlight the complexity of birth weight regulation and the need for larger and more diverse populations in future studies to identify universally significant loci.

Among the SNPs identified in this study, CNCB10001505 and CNC10180515 exhibit robust and consistent genotype effects on birth weight, while CNC10160101 and CNC10170045 display population-dependent trends. The remaining six loci reinforce the importance of genotype-dependent effects on birth weight, with consistent trends across populations, further contributing to our understanding of the genetic architecture underlying birth weight in pigs. CNCB10001505 and CNC10160101 in particular have the potential to be integrated into marker-assisted selection programs to improve birth weight in pigs. MAS allows for the selection of animals with desirable genetic markers at an early stage, thereby accelerating genetic progress and improving production efficiency. However, the low frequency of favorable alleles at these loci in both the discovery and validation populations suggests that careful management of genetic diversity will be necessary to avoid inbreeding and maintain genetic variation in breeding programs.

Future research should focus on functional validation of the candidate genes identified in this study, particularly *MARCHF11*, *NFATC3*, and *SGCD*, to elucidate their roles in fetal development and growth. Additionally, expanding the sample size to include more pig populations and breeds will help to confirm the generalizability of these findings. The integration of genomic data with environmental factors in large-scale studies will provide a more comprehensive understanding of the genetic and environmental interactions that influence birth weight in pigs, ultimately leading to more effective and sustainable breeding strategies.

## 5. Conclusions

This study provides valuable insights into the genetic basis of birth weight in pigs. In this genome-wide association study of Landrace pigs, we identified seven significant SNPs and 13 candidate genes associated with birth weight. These genes are enriched in the biological process of developmental processes (GO:0032502) and muscle tissue development (GO:0060537), suggesting their potential roles in regulating birth weight. Validation in an expanded population confirmed two novel loci, CNCB10001505 and CNC10160101, and identified a new candidate gene, *MARCHF11*, significantly associated with birth weight. These findings enhance our understanding of the genetic architecture influencing birth weight in pigs and provide valuable targets for marker-assisted selection in breeding programs. Future research should focus on functional validation of these candidate genes, exploration of gene–environment interactions, and cross-validation in diverse pig breeds to further elucidate the complex genetic mechanisms underlying birth weight.

## Figures and Tables

**Table 1:** Statistical description of birth weight phenotypes in different pig populations.

Species	Count	Mean	SD ^1^	Median	Min	Max	CV ^2^ (%)
Landrace	1125	1.42	0.20	1.40	0.80	2.20	13.73
Yorkshire	998	1.22	0.25	1.22	0.70	2.20	20.66
Total	2123	1.33	0.24	1.30	0.70	2.20	18.34

^1^ SD means standard deviation; ^2^ CV means coefficient of variation.

**Table 2:** Environmental and animal factors affecting birth weight in different pig populations.

Population	Breeds	Herd	Year	Season	Parity	Sex
Landrace	--	**	**	***	***	***
Yorkshire	--	***	***	ns	ns	--
Total	***	***	*	***	ns	***

Significance markers: *** represents *p* ≤ 0.001, ** represents *p* ≤ 0.01, * represents *p* ≤ 0.05, and ns represents no statistical significance (*p* ≥ 0.05).

**Table 3:** SNPs and candidate genes associated with birth weight in Landrace pigs.

SNP	Chr. ^1^	Position/bp	Allele	*p*-Value	Candidate Gene
CNC10010096	1	4,264,479	A/G	1.90 × 10^−7^	--
CNCB10001505	1	307,515,291	T/C	6.21 × 10^−5^	--
CNC10020434	2	18,828,505	A/G	1.81 × 10^−7^	*TTC17*
CNC10032309	3	117,474,270	T/G	2.07 × 10^−7^	*LDAH*
CNC10032128	3	109,118,738	T/C	4.00 × 10^−7^	--
CNC10042365	4	125,588,650	G/A	2.25 × 10^−5^	*HFM1*
CNCB10003289	4	52,145,174	G/A	3.89 × 10^−5^	*RALYL*
CNC10051462	5	78,270,522	A/G	6.53 × 10^−7^	*--*
CNC10060592	6	28,761,105	T/A	5.73 × 10^−9^	*NFATC3*
CNC10061395	6	66,110,498	T/C	3.58 × 10^−5^	--
CNC10082700	8	133,591,911	C/A	5.04 × 10^−7^	--
CNCB10009227	13	159,079,583	T/C	1.25 × 10^−5^	*CMSS1*
CNCB10009475	13	200,184,861	T/C	4.26 × 10^−5^	*CLDN14*
CNCB10009612	14	6,581,366	G/A	3.57 × 10^−6^	*PIWIL2*
CNCB10011512	16	66,786,864	T/G	1.43 × 10^−6^	*SGCD*
CNC10160101	16	5,379,864	C/T	1.52 × 10^−6^	*MARCHF11*
CNCB10011490	16	61,520,473	T/C	6.53 × 10^−5^	*GABRG2*
CNC10161130	16	57,760,728	A/G	9.15 × 10^−5^	*TENM2*
CNC10170045	17	2,300,590	T/C	6.04 × 10^−5^	*SGCZ*
CNC10180515	18	26,725,722	G/A	3.16 × 10^−5^	--

^1^ Chr. represents Chromosome.

## Data Availability

The dataset used and analyzed during the current study is available from the corresponding author upon reasonable request.

## Abbreviations

The following abbreviations are used in this manuscript:

GWAS	Genome-wide association study
MAS	Marker-assisted selection
LL	Landrace pigs
YY	Yorkshire pigs
LSM	Least-squares means
MAF	Minor allele frequency
PCA	Principal component analysis
FarmCPU	Fixed and Random Model Circulating Probability Unification
GO	Gene Ontology
KW test	Kruskal–Wallis test
